# Arsenite exposure induces premature senescence and senescence-associated secretory phenotype (SASP) in human hepatocyte-derived cell line Huh-7

**DOI:** 10.1265/ehpm.24-00139

**Published:** 2024-12-27

**Authors:** Kazuyuki Okamura, Miyuki Sato, Takehiro Suzuki, Keiko Nohara

**Affiliations:** Health and Environmental Risk Division, National Institute for Environmental Studies, Tsukuba 305-8506, Japan

**Keywords:** Arsenic, Cellular senescence, SASP, Cancer, Liver, Hepatocellular carcinoma, Gene expression, Environment

## Abstract

**Background:**

Chronic arsenite exposure has been known to induce cancer in various organs; however, the underlying mechanisms remain elusive. The characteristic feature of carcinogenesis due to arsenic exposure is that the disease develops after a prolonged latent period, even after cessation of exposure. Our previous study revealed that arsenite exposure induces premature senescence in hepatic stellate cells and suggests that the senescence-associated secretory phenotype (SASP) factors from the senescent cells promote hepatic carcinogenesis. However, arsenite exposure in the liver occurs not only in hepatic stellate cells, but also in hepatocytes. Therefore, we examined whether arsenite exposure in hepatocytes also causes premature senescence and the enhancement of SASP factors. We also assessed whether those effects remained after cessation of arsenite exposure.

**Methods:**

Human hepatocyte-derived cell line Huh-7 was exposed to sodium arsenite for 72 hours to determine the concentration at which cell proliferation was inhibited. In the 5 µM of exposure, various cellular senescence markers and SASP factors were analyzed and compared with unexposed cells. We also examined whether those senescence markers and SASP factors were maintained after cessation of arsenite exposure. Finally, we explored whether the increased expression of SASP factor, which was upregulated in hepatocytes by arsenic exposure in this study, is related to the prognosis of human hepatocellular carcinoma.

**Results:**

After exposure to 5 µM of sodium arsenite for 72 hours, various senescent features, such as the induction of *P21* mRNA, the reduction of *LAMINB1* mRNA, morphological changes, phosphorylation of P53, and the presence of SA-β-gal positive cells were observed. Those changes were maintained after cessation of arsenite exposure. In addition, mRNA levels of SASP factors (*MMP1*, *MMP3*, *MMP10*, *GDF15*, *PAI-1*, and *IL-6*) were increased after arsenite exposure, and their high expression levels were maintained after cessation of arsenite exposure. Furthermore, by analyzing the TCGA database, we found that the increased expression levels of many SASP factors negatively correlated with prognosis.

**Conclusions:**

Arsenite exposure induces premature senescence in hepatocyte-derived cells and increases SASP factors that are related to hepatic tumorigenesis. Once arsenite exposure induces premature senescence, the senescent cells remain even after cessation of exposure.

**Supplementary information:**

The online version contains supplementary material available at https://doi.org/10.1265/ehpm.24-00139.

## Graphical Abstract

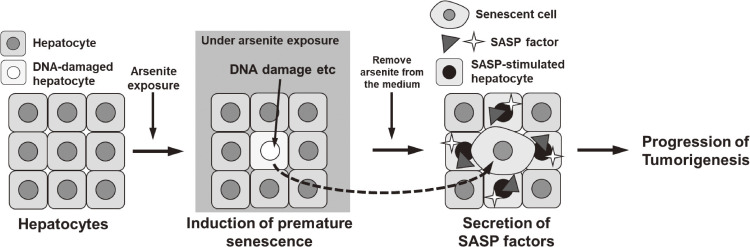

## 1. Introduction

Naturally-derived inorganic arsenic is found in contaminated ground water. People who drink such water by using tube wells are exposed to arsenite [1]. Chronic arsenic poisoning is caused by chronic ingestion of water containing arsenic and has been found to cause cancer of the skin, lungs, and liver [2, 3]. In addition, carcinogenesis is known to occur after an incubation period even after cessation of arsenic exposure [4]. The mechanisms by which arsenic exposure induces carcinogenesis are still elusive.

Cellular senescence was first observed by Hayflick and Moorhead in 1961 [5]. They showed that cells can only divide a limited number of times, which they found was caused by telomere shortening during each cell division. That in turn leads to a critical telomere length which triggers a permanent arrest in growth. This type of senescence is termed replicative senescence. Cellular senescence is also caused by various stresses, independent of normal telomere shortening, and is termed premature senescence [6, 7]. Traditionally, since cellular senescence is a permanent, non-proliferative state, the phenomenon has been thought to have tumor-suppressive properties [8]. In the last decade, however, it has become clear that if senescent cells continue to exist for a long period of time without being eliminated, they contribute to the promotion of carcinogenesis through the secretion of inflammatory cytokines and other phenomena known as senescence-associated secretory phenotype (SASP) [9–11]. Since many reports have shown that fibroblast senescence contributes to tumorigenesis [10, 12, 13], our previous study focused on hepatic stellate cells, fibroblasts in the liver, and showed that arsenite exposure induces premature senescence in hepatic stellate cells and SASP such as IL-8 and MMP1, which are associated with tumorigenesis [14]. When humans are exposed to arsenic in the environment, however, not only hepatic stellate cells but also hepatocytes, the major parenchymal cells in the liver, are exposed to arsenic. Therefore, it is assumed that premature senescence might be induced in hepatocytes by arsenite exposure and might be involved in carcinogenesis. Recent reports have shown that cellular senescence of hepatocytes also contributes to tumorigenesis [15–17]. In this study using human hepatocyte-derived cell line Huh-7, we investigated whether arsenite exposure induces premature senescence in hepatocytes followed by SASP induction. We also examined whether the feature of arsenite-induced premature senescence and SASP factors were maintained even after cessation of the exposure.

## 2. Materials and methods

### 2.1. Cell culture

The human hepatocyte-derived cell line, Huh-7, purchased from the JCRB Cell Bank (JCRB0403), was cultured in DMEM (Sigma D6046 or Fujifilm Wako Pure Chemical Corporation 04129775) with 10% FBS and 1% penicillin-streptomycin at 37 °C in a humidified incubator in the presence of 5% CO_2_.

### 2.2. Arsenite treatment

The compound sodium arsenite (NaAsO_2_), acquired from Sigma (product number 228699), was dissolved in saline solution, either at 10 mM or 100 mM concentrations, and then passed through a 0.22-µm filter. Portions of this solution were appropriately diluted in distilled water and added to the medium. Specific concentrations of sodium arsenite used in each experiment are detailed in the section Materials and Methods.

### 2.3. Cell proliferation assay

We conducted the RealTime-Glo™ MT Cell Viability Assay (Promega, G9711) in accordance with the guidelines provided by the manufacturer. In brief, 2500 cells were placed into a 96-well flat clear-bottomed white polystyrene TC-treated microplate (Corning, 3610) and subjected to exposure with or without arsenite at varying concentrations (0–100 µM). The luminescence levels were recorded from 0 to 72 hours post-arsenite exposure using a GloMax^®^ Discover Microplate Reader with 0.5 s. In experiments measuring changes in cell proliferation after discontinuing arsenic exposure, cells were cultured for 72 hours in the presence or absence of 5 µM sodium arsenite. After the culture, cells were harvested and counted, then 2500 of them were reseeded into each well and subsequent culture. The luminescence levels were recorded after 4, 24, 48, and 72 hours.

Furthermore, an alternative technique was utilized to evaluate cell proliferation. The CellTiter-Glo^®^ 2.0 reagent (Promega, G9242) was employed at 24 hours or 72 hours post-exposure to arsenite. In brief, 2500 cells were seeded into a 96-well flat clear-bottomed white polystyrene TC-treated microplate (Corning, 3610) and treated with varying concentrations of arsenite (0–100 µM) in 100 µl medium. After a 24- or 72-hour incubation period, 100 µl of CellTiter-Glo^®^ 2.0 reagent (Promega, G9242) was added to each well and gently mixed at 22 °C for 2 minutes at 500 rpm using ThermoMixer C (Eppendorf 5382000023). Following a 10-minute incubation at room temperature, luminescence intensities were measured using a GloMax^®^ Discover Microplate Reader (GM3000) with 0.5-second integration.

### 2.4. Quantitative real-time RT PCR

Quantitative real-time RT-PCR was performed as previously described [14]. The cells were utilized for the extraction of total RNA using the RNeasy Mini Kit (Qiagen) following the recommended procedure. The concentration of RNA was determined by employing a NanoDrop ND-1000 spectrophotometer (Thermo Fisher Scientific, USA). Subsequently, RNA (100 ng) was converted into complementary DNA (cDNA) through reverse transcription using an RNA PCR kit (AMV) Ver. 3.0 (Takara). Quantitative real-time PCR analysis was carried out utilizing the Light Cycler 480 Real-Time PCR System (Roche Diagnostics). The PCR blend consisted of LightCycler^®^480 SYBR Green I Master, 0.2 µM of each forward and reverse primer, and cDNA (equivalent to 2 ng total RNA). The PCR process involved an initial denaturation at 95 °C for 10 minutes, followed by 50 cycles at 95 °C for 10 seconds, 64 °C or 68 °C for 10 seconds, and 72 °C for 10 seconds, with a single fluorescence measurement. A melting curve program (69 °C to 95 °C with a heating rate of 0.1 °C/s and continuous fluorescence measurement) was implemented, followed by a cooling step to 40 °C. For the assessment of amplifications in the experimental samples during the log-linear phase, Light Cycler quantification software (Version 1.5.1.62) was employed, using the standard curve derived from a dilution series of control cDNA as a reference. Control cDNA was generated by combining samples from the control and arsenite-exposed groups in each exposure experiment. The levels of cellular gene expression, including *P21*, *LAMINB1*, *MMP1*, *MMP3*, *MMP10*, *GDF15*, *PAI-1*, *VEGFA*, and *IL-6* were quantified, with *18S rRNA* or *RPLP1* utilized as the internal reference gene. Details regarding the primer sequences and annealing temperatures can be found in Table S1. The relative expression level of each gene was normalized to the expression level of *18S rRNA* or *RPLP1*.

### 2.5. Observation of morphological features

Cells were grown in a solution with either no sodium arsenite or 5 µM of sodium arsenite in 10-cm dishes. After 72 hours, the cells were examined using a microscope (Olympus, IX70) at a magnification of 100×.

### 2.6. Western blotting

Western blotting was done as previously described with some modifications [18]. Briefly, cells were lysed with RIPA buffer (Nacalai Tesque, 16488-34) with complete inhibitor cocktail (Roche) and sonicated using a Bioruptor (Cosmo Bio, UCD-200TM) (“high” setting for 5 minutes with 30 s “ON” and 30 s “OFF” cycle). The protein concentrations were determined using a bicinchoninic acid protein assay kit (Pierce, 23225). All samples were denatured in SDS sample buffer (50 mM Tris-HCl pH 6.8, 2% SDS, 10% glycerol, 100 mM DTT, 0.001% bromophenol blue) at 95 °C for 5 minutes. The denatured proteins were separated using SDS polyacrylamide gel electrophoresis and transferred on to a 0.45-µm PVDF membrane (Amersham). The membranes were blocked with 4% BSA in PBS (-) and incubated at room temperature for 1 hour with the following primary antibodies: anti-P-P53 (Ser15) (1:1000, Cell Signaling, #9284), anti-P53 (1:1000, Santa Cruz, sc-126) and anti-β-actin (1:10000, Sigma, A5441). Thereafter, the membranes were incubated at room temperature for 1 hour with the following secondary antibodies: horseradish peroxidase-conjugated anti-rabbit IgG (1:10000, Sigma, A6154) or horseradish peroxidase-conjugated anti-mouse IgG (1:10000, Sigma, A4416). The ECL Prime western blotting Detection Reagent (Amersham, RPN2232) and Versa doc (MP5000 system, Bio-Rad) were used for the visualization and quantification of the signal.

### 2.7. SA-β-gal staining

SA-β-gal staining was conducted following the protocol outlined by Debacq-Chainiaux et al. (2009), as referenced in an earlier study [19]. SA-β-gal is defined as β-galactosidase activity detectable at pH 6.0 in senescent cells, which can distinguish between senescent and non-senescent cells [20]. Briefly, cells were exposed for specific time intervals, washed twice with PBS (-), and then fixed with 2% (vol/vol) formaldehyde and 0.2% (vol/vol) glutaraldehyde for 5 minutes. Subsequently, cells underwent triple washing with PBS (-) and were subjected to SA-β-gal staining solution in a humidified 37 °C incubator without CO_2_ for 16–17 hours. Microscopic examination (Olympus, IX70) at 200× magnification was employed for cell observation. Quantification involved manual counting of stained cells among randomly selected 5 fields of view.

### 2.8. Immunofluorescent staining

Cells were cultured in a medium containing 0, 5 or 10 µM of sodium arsenite in 3.5-cm dishes for 72 hours. After the culture, the medium was removed and 4% PFA was added, followed by a 15-min incubation. After the incubation, cells were washed with PBS (-) three times, and Triton-X100 was added, followed by 15 min of incubation. After the incubation, cells were washed with PBS (-) three times, and added to a blocking buffer (Nacalai, 06349-64) followed by 1 hour incubation. After the incubation, the blocking buffer was removed and anti-γ-H2AX (Millipore 05-636) or anti-MMP3 antibody (abcam ab52915) was added, followed by overnight incubation at 4 °C. After the incubation, cells were washed with PBS-T three times, and secondary antibodies (Invitrogen A32723 or A32731) were added, followed by 1 hour incubation at room temperature. After the incubation, cells were washed with PBS-T three times, and Hoechst 33342 solution (Dojindo, H342) was added, followed by 15 minutes of incubation in a 37 °C CO_2_ incubator. After the incubation, cells were washed with PBS (-) three times, and the cells were observed using a fluorescence microscope (Keyence BZ-X710).

### 2.9. Analysis of the expression profiles of genes in human liver cancer

As previously described [14], data on gene expression patterns in hepatocellular carcinoma were acquired from the Human Protein Atlas (https://www.proteinatlas.org/humanproteome/pathology). It involved the analysis of transcription data sourced from The Cancer Genome Atlas (TCGA), specifically from 365 liver cancer patients (119 females and 246 males), with most patients (235 individuals) being alive during the data collection period. Specifically, we retrieved expression information pertaining to the SASP factor gene from the pathology section. The SASP factors we searched for in this study were upregulated in response to arsenite exposure in Huh7 cells. We examined the relationship between their expression and prognosis.

### 2.10. Statistical analysis

To assess the statistical significance of variances between the two groups, a two-tailed unpaired Student’s t-test was applied. For comparisons involving more than two groups, the Tukey-Kramer test was utilized. To examine the association between mRNA expression levels and patient survival in human HCC data, the log-rank test was employed. All tests were deemed statistically significant at a threshold of P < 0.05.

## 3. Results

**Fig. 1:**
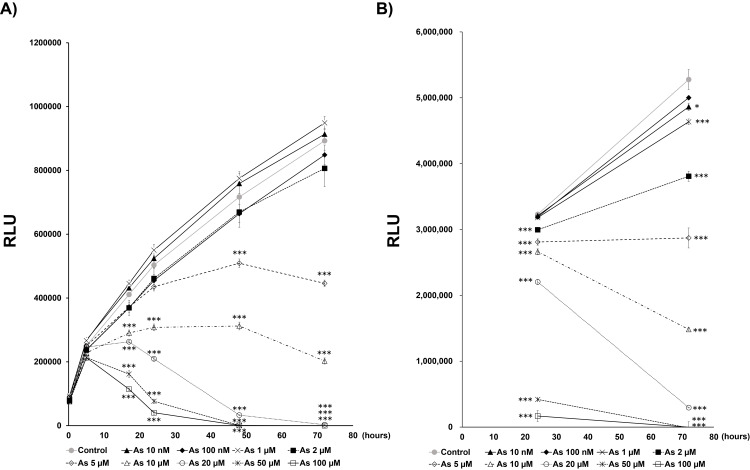
The arsenite concentration at which cell proliferation was inhibited A) Time dependent changes of cell number after arsenite exposure. The cell viability was assayed using Real-time Glo^®^ in Huh-7 cells. Data are presented as the means ± SEM (n = 3). RLU: relative luminescence units. The Tukey-Kramer test was used for statistical analysis. ***significantly different from the control. ***P < 0.001. B) The cell viability after arsenite exposure at the typical time points (24 h and 72 h) was assayed using Cell-Titer Glo^®^ in Huh-7 cells. Data are presented as the means ± SEM (n = 3). RLU: relative luminescence units. The Tukey-Kramer test was used for statistical analysis. *, ***significantly different from the control. *P < 0.05, ***P < 0.001.

Since cellular senescence is a phenomenon in which cells no longer divide, even when stimulated by a growth factor, we first evaluated the dose of arsenite exposure that induces suppression of cell proliferation in human hepatoma cell line Huh-7. We exposed the cells to 0–100 µM of sodium arsenite and evaluated the change in cell proliferation. After 72 hours of culture, cell proliferation was significantly suppressed following arsenite exposure at concentrations above 5 µM (Fig. 1A). Another method for evaluating cell proliferation showed that, after 5 µM of exposure, the cell number was similar after both 24 hours and 72 hours (Fig. 1B). We therefore decided to examine in detail whether premature senescence is induced around the concentration of 5 µM.

**Fig. 2:**
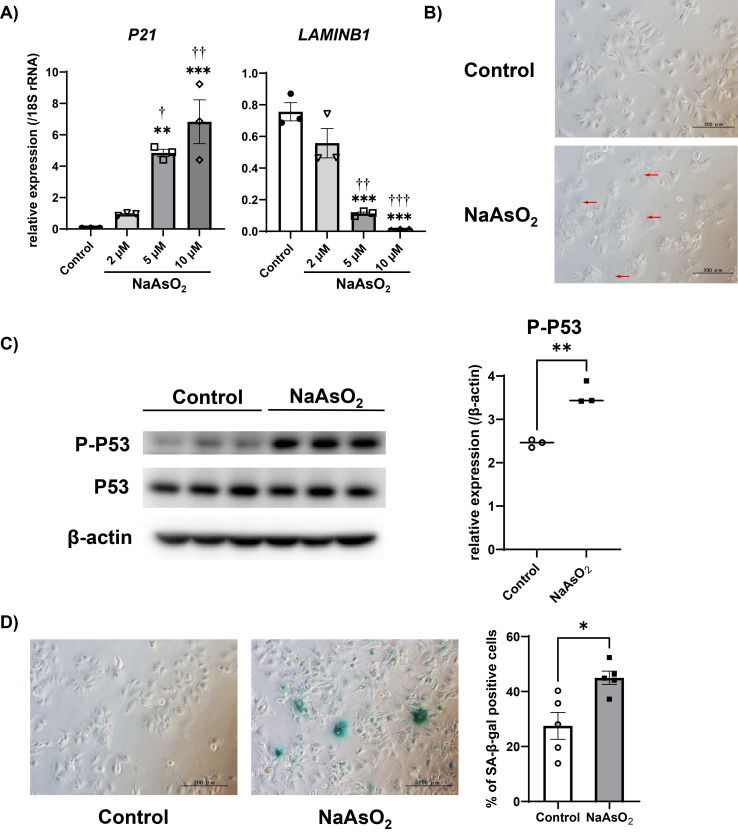
Arsenite exposure induced premature senescence in the Huh-7 cells A) Changes in the gene expressions of senescence markers in the Huh-7 cells after arsenite exposure. The mRNA level of each gene was quantified by real-time PCR and normalized to the expression level of 18S rRNA. The bar graph shows the relative gene expression levels of each senescence marker after 72 hours of arsenite exposure. Data are presented as the means ± SEM (n = 3). The Tukey-Kramer test was used for statistical analysis. **, ***significantly different from the control; †, ††, †††significantly different from 2 µM. †P < 0.05, **, ††P < 0.01, ***, †††P < 0.001. B) Representative microscopic photographs of Huh-7 cells after 5 µM of arsenite exposure for 72 hours. The red arrowheads indicate cells exhibiting the typical morphological changes of cellular senescence. Scale bars are 200 µm. C) The protein levels of P-P53 (Ser 15) and β-actin detected by Western blotting after 72 hours of 5 µM arsenite exposure (left panel). The protein expression level was normalized to that of β-actin (right panel). Data are presented as the means ± SEM (n = 3). The two-tailed unpaired Student’s t-test was used for statistical analysis. **significantly different from the control; **P < 0.01. D) Representative picture of SA-β-gal staining of Huh-7 cells after 72 hours of arsenite exposure (left panel). % of SA-β-gal positive cells were calculated from five representative views (right panel). The two-tailed unpaired Student’s t-test was used for statistical analysis. *significantly different from the control; *P < 0.05.

To investigate whether arsenite exposure induces premature senescence in Huh-7 cells, we quantified mRNA levels of senescence markers (P21 and LAMINB1) after arsenite exposure for 72 hours. Consistent with senescence features, mRNA level of *P21* was increased and *LAMINB1* was decreased, by 5 and 10 µM sodium arsenite exposure, respectively (Fig. 2A). Since we observed those mRNA changes starting at 5 µM of exposure, we further evaluated other senescence markers at exposures of 5 µM. Cell morphological changes such as enlargement and flattening were observed after arsenite exposure (Fig. 2B), and the protein level of phospho-P53 (Ser15) was also increased after arsenite exposure (Fig. 2C). Furthermore, the ratio of SA-β-gal positive cells was increased by arsenite exposure (Fig. 2D). These results suggested that arsenite exposure induces premature senescence in Huh-7 cells.

**Fig. 3:**
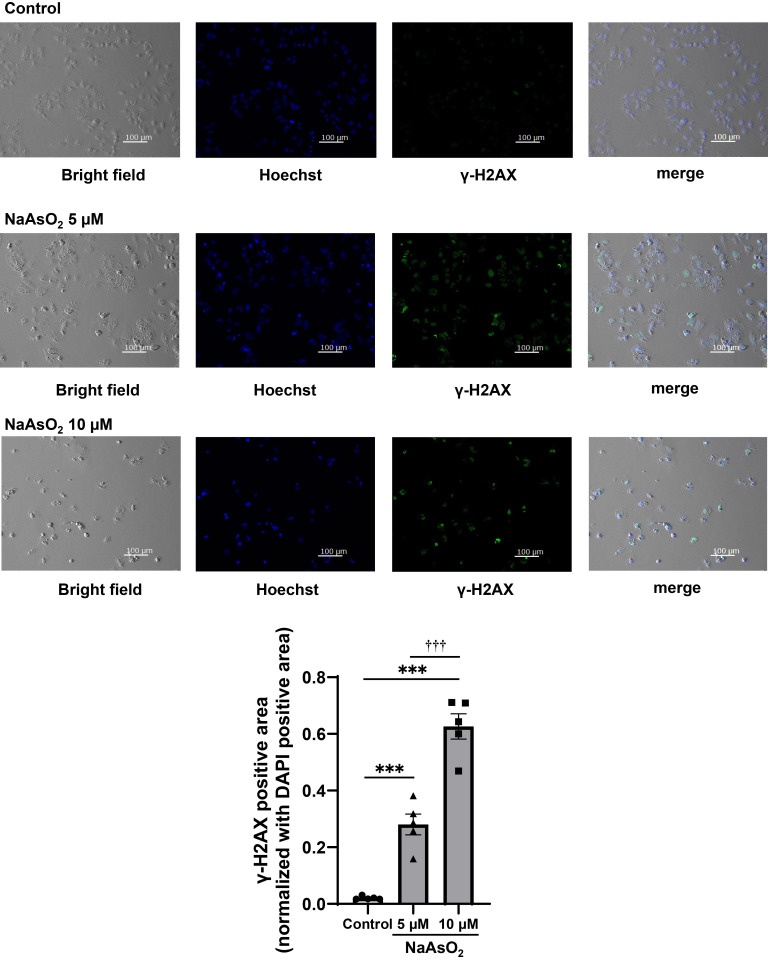
Arsenite exposure induced DNA double strand breaks in the Huh-7 cells Representative bright field images and immunofluorescence staining images of γ-H2AX and Hoechst 33342 in Huh-7 cells with or without 75 hours of sodium arsenite exposure. Leftmost images show the bright field, the second from left images show the Hoechst that reflect nuclei, the third from left images show γ-H2AX, and the rightmost images show the merge of those left three images. Quantitative result using data from 5 randomly selected fields of view (bottom panel). The bottom panel shows the value of γ-H2AX-positive area/field normalized with DAPI-positive area. The Tukey-Kramer test was used for statistical analysis. ***significantly different from the control; †††significantly different from 5 µM. ***, †††P < 0.001.

Since our previous studies suggested that arsenite exposure causes DNA damage that can induce premature senescence [14, 19], we observed γ-H2AX expression level as DNA double-strand break marker by using immunofluorescence staining. As a result, the high intensity of γ-H2AX was observed after 5 or 10 µM sodium arsenite exposure (Fig. 3). These results suggested that arsenite exposure induces DNA damage that can cause premature senescence.

**Fig. 4:**
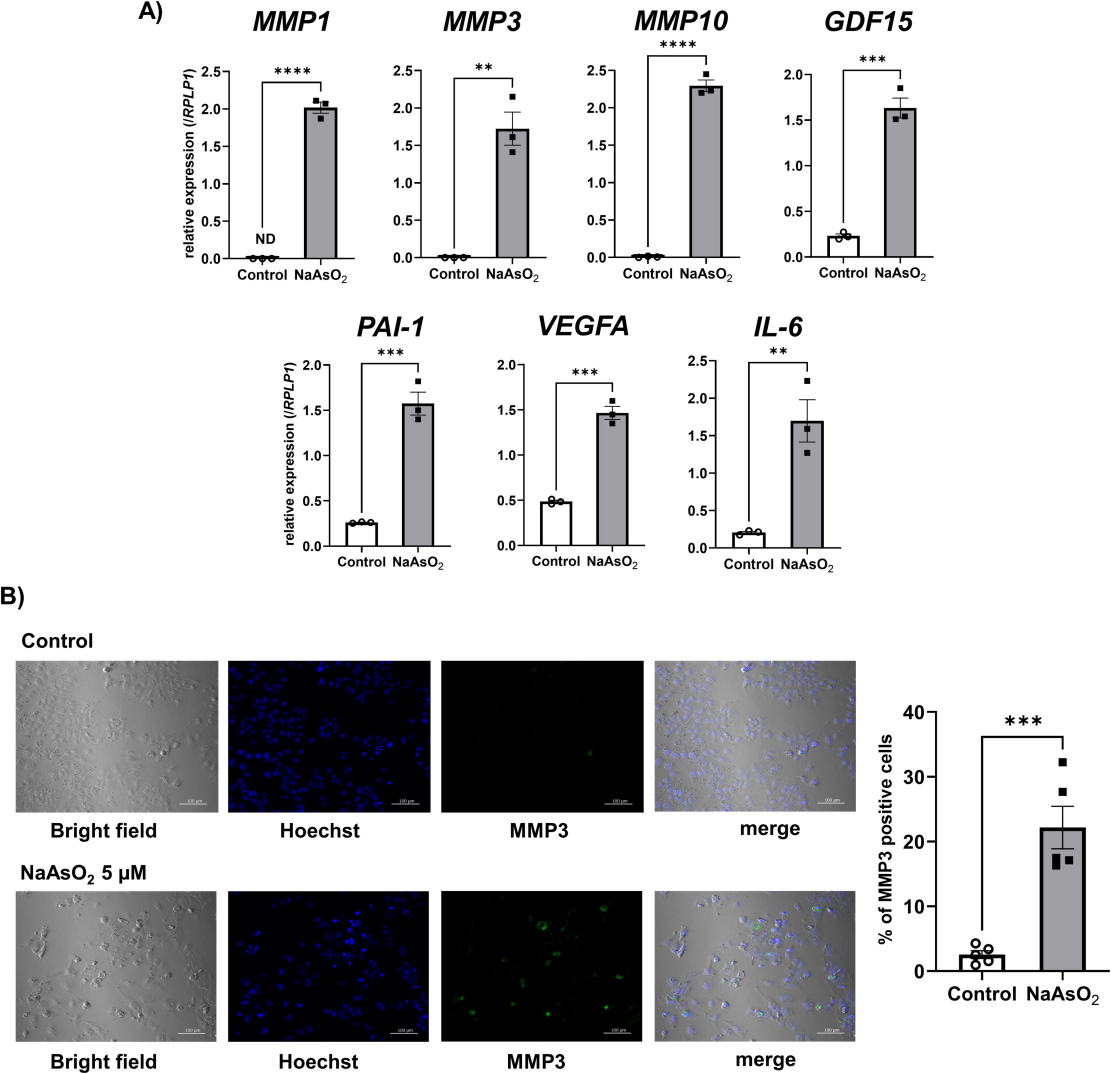
Arsenite exposure in Huh-7 cells upregulated genes encoding SASP factors A) Changes in the gene expressions of SASP factors in the Huh-7 cells after arsenite exposure. The mRNA expression level of each gene was quantified by real-time PCR and normalized to the expression level of RPLP1. The bar graph shows the relative gene expression level of each SASP factor after 72 h of arsenite exposure. Data are presented as the means ± SEM (n = 3). The two-tailed unpaired Student’s t-test was used for statistical analysis. **, ***, ****significantly different from the control; **P < 0.01, ***P < 0.001, ****P < 0.0001. B) Representative bright field images and immunofluorescence staining images of MMP3 and Hoechst 33342 in Huh-7 cells with or without 75 hours of sodium arsenite exposure (left panel). Quantitative result using data from 5 randomly selected fields of view (right panel). The two-tailed unpaired Student’s t-test was used for statistical analysis. ***significantly different from the control; ***P < 0.001.

Next, we investigated whether SASP factors were upregulated by arsenite exposure when cells suffered premature senescence from the exposure. We quantified mRNA levels of SASP factors by qPCR. All SASP factors we examined in the current experiment, such as *MMP1*, *MMP3*, *MMP10*, *GDF15*, *PAI-1*, *VEGFA*, and *IL-6* were significantly upregulated by arsenite exposure (Fig. 4A). Among those mRNA increased SASP factors, we confirmed an increase of protein level of MMP3 by immunofluorescence staining consistent with mRNA expression change (Fig. 4B).

Based on these results, we suggest that arsenite exposure induces premature senescence in Huh-7 cells.

**Fig. 5:**
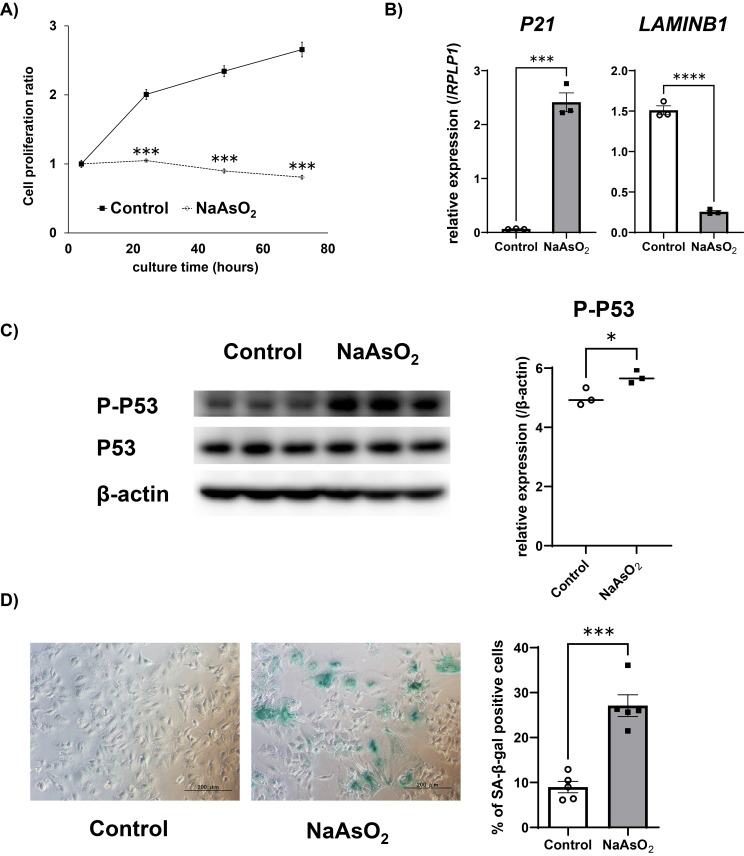
Arsenite-induced premature senescence features persisted after exposure cessation in Huh-7 cells A) Time dependent changes of cell proliferation ratio (the value after 4 hours of incubation was set to 1) after cessation of arsenite exposure. The relative cell viability was assayed using Real-time Glo^®^ in Huh-7 cells. Data are presented as the means ± SEM (n = 3). RLU: relative luminescence units. The two-tailed unpaired Student’s t-test was used for statistical analysis. ***significantly different from the control; ***P < 0.001. B) Changes in the gene expressions of senescence markers in the Huh-7 cells after arsenite exposure. The mRNA level of each gene was quantified by real-time PCR and normalized to the expression level of RPLP1. The bar graph shows the relative gene expression levels of each senescence marker after 72 hours of arsenite exposure followed by 100 hours of culture in the medium without arsenite. Data are presented as the means ± SEM (n = 3). The two-tailed unpaired Student’s t-test was used for statistical analysis. ***, ****significantly different from the control. ***P < 0.001, ****P < 0.0001. C) The protein levels of P-P53 (Ser 15), P53 and β-actin detected by Western blotting after 72 hours of arsenite exposure followed by 100 hours of culture in the medium without arsenite (left panel). The protein expression level was normalized to that of β-actin (right panel). Data are presented as the means ± SEM (n = 3). The two-tailed unpaired Student’s t-test was used for statistical analysis. *significantly different from the control. *P < 0.05. D) SA-β-gal staining of Huh-7 cells after 72 hours of arsenite exposure followed by 7 days of culture in the medium without arsenite (left panel). % of SA-β-gal positive cells were calculated from five representative views (right panel). The two-tailed unpaired Student’s t-test was used for statistical analysis. ***significantly different from the control; ***P < 0.001.

Since arsenic exposure-induced carcinogenesis is known to occur after an incubation period even after cessation of arsenic exposure, we next examined whether arsenite exposure-induced premature senescence and increased-SASP factors were maintained even after cessation of the exposure. After exposure to 5 µM of sodium arsenite for 72 hours, followed by additional cell culture without arsenite for 72 hours, the cell proliferation ratio was not changed when cells were exposed to arsenite prior to culture (Fig. 5A). In addition, gene expression level of *P21* was upregulated and *LAMINB1* was downregulated after 100 hours culture without arsenite following 72 hours of arsenite exposure (Fig. 5B). At the same time, the high protein expression level of phosphor-P53 (Ser15) was maintained (Fig. 5C). Moreover, other senescence markers such as morphological changes and appearance of SA-β-gal positive cells were also prominently observed after 7 days of culture without arsenite following 72 hours of arsenite exposure (Fig. 5D). These results suggest that arsenite exposure-induced premature senescence is maintained even after cessation of arsenite exposure.

**Fig. 6:**
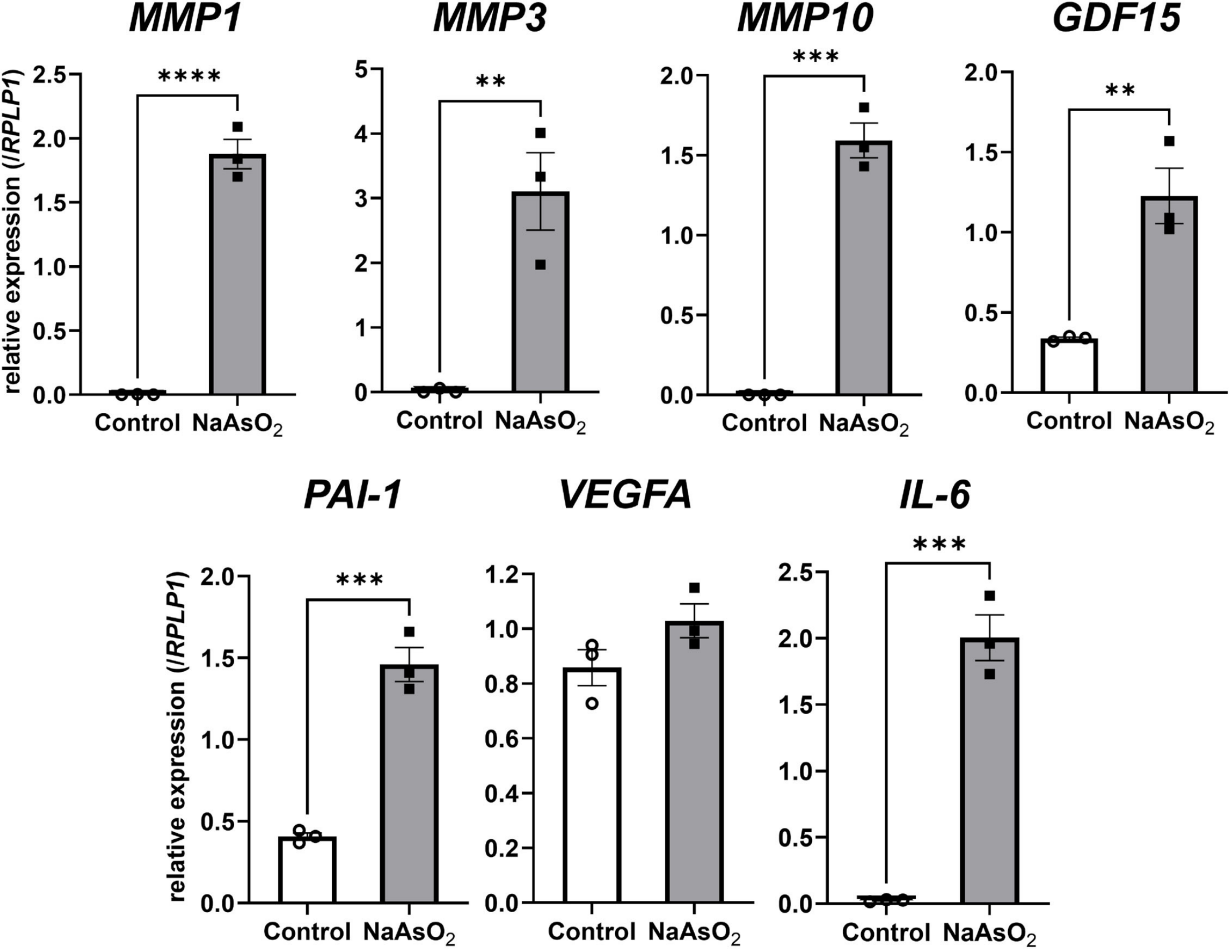
Arsenite-induced expression levels of SASP factors persisted after exposure cessation in Huh-7 cells Changes in the expression levels of genes encoding the SASP factors in the Huh-7 cells following cessation of arsenite exposure after 72 hours. The mRNA expression level of each gene was quantified by real-time PCR and normalized to the expression level of *RPLP1*. The bar graph shows the relative gene expression level of each SASP factor at 100 hours following cessation of 72 hours of arsenite exposure. Data are presented as the means ± SEM (n = 3). The two-tailed unpaired Student’s t-test was used for statistical analysis. **, ***, ****significantly different from the control, **P < 0.01, ***P < 0.001, ****P < 0.0001.

Next, to investigate whether SASP factors that were induced by arsenite exposure were increased even after cessation of arsenite exposure, we quantified the gene expression levels of SASP factors after 100 hours cell culture without arsenite following 72 hours of arsenite exposure. Among the SASP factors we examined, all the SASP factors without *VEGFA* were highly expressed when cells were exposed to arsenite prior to culture without arsenite (Fig. 6). These results suggest that arsenite exposure-induced SASP factors were also maintained even after cessation of arsenite exposure, at least in part.

**Fig. 7:**
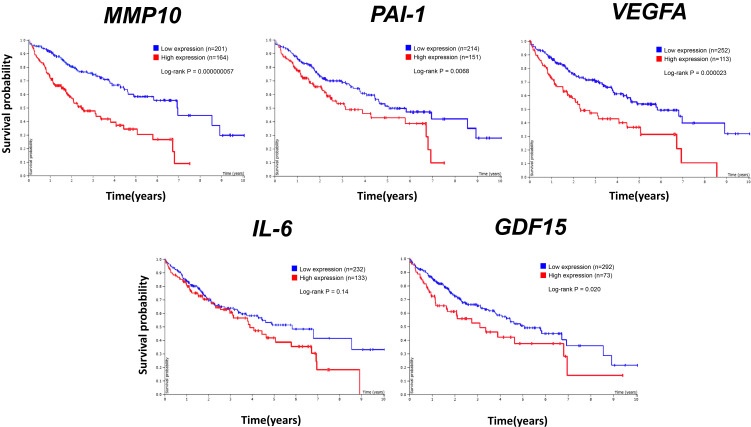
SASP factors upregulated by arsenite exposure correlate with poor liver cancer prognosis in patients Kaplan-Meier curves for overall survival in liver cancer patients with higher and lower expression levels of *MMP10*, *PAI-1*, *VEGFA*, *IL-6*, *GDF15*. Data were obtained from the Human Protein Atlas (https://www.proteinatlas.org/humanproteome/pathology). *MMP10*, *PAI-1*, *VEGFA*, *IL-6* and *GDF15* are SASP factors that were upregulated in response to arsenite exposure in the Huh-7 cells. The thresholds for higher and lower expressions were set automatically.

Finally, to investigate whether arsenite-induced SASP factors are related to tumorigenesis, we analyzed the relationship between gene expression levels of the SASP factors and poor prognosis in liver cancer from the Human Protein Atlas website. Among the examined genes, all the genes except *IL-6* showed significant correlation between gene expression level and poor prognosis (Fig. 7). *MMP1* and *MMP3* expression levels were already shown to be significantly related to poor prognosis in our previous study [14]. These results suggest that arsenite exposure-induced SASP factors in hepatocytes have the potential to lead to progression of liver cancer.

## 4. Discussion

Our previous study showed that arsenite exposure induces premature senescence in hepatic stellate cells which then contribute to development of liver tumorigenesis [14]. However, not only hepatic stellate cells but also hepatocytes are exposed to arsenite if humans are exposed to arsenite. In the present study, we revealed that at least some population of hepatocytes have the potential to induce premature senescence from arsenite exposure and to enhance SASP factors that contribute to poor prognosis. Importantly, once premature senescence was induced by arsenite exposure, the feature of senescent cells and enhanced SASP factors were maintained even after cessation of arsenite exposure. This phenomenon led us to consider that arsenite-induced premature senescence followed by SASP induction can play an important role in the mechanism of arsenite-induced carcinogenesis, which appears even after cessation of arsenite exposure. While our study was in progress, Aki et al. [21] reported that arsenic exposure induced premature senescence in Huh-7 cells. The present study further clarified that many SASP factors, which were increased by the exposure, and the increase in those SASP factors were related to poor prognosis in HCC. Importantly, the senescence features and increase of SASP factors remained after cession of the arsenite exposure. These results suggest that premature senescence induced by arsenite exposure and the subsequent increase in SASP factors can contribute to the tumorigenic mechanism of arsenite, which develops after a latent period.

Since various mechanisms, including oxidative stress, DNA damage and repair, and epigenetic modifications have been reported to be involved in arsenic-induced tumorigenesis [22], further studies are needed to clarify the contribution of those factors and SASP to the tumorigenesis.

In general, cellular senescence acts to suppress aberrant proliferation of the cells. If, however, senescent cells continue to exist for a long period of time without being eliminated, they contribute to the promotion of carcinogenesis through the secretion of inflammatory cytokines and other phenomena known as senescence-associated secretory phenotype (SASP) [9–11]. Our previous study using hepatic stellate cells showed that arsenite exposure increases SASP factors such as *MMP1*, *MMP3* and *IL-1β* [14]. Among them, MMP1 and MMP3 are known to be associated with cancer growth, invasion, and metastasis [23–25], and they were also increased in Huh7 cells after arsenite exposure. In addition, high expression levels of all those genes were significantly related to poor prognosis by analyzing the Human Protein Atlas (https://www.proteinatlas.org/humanproteome/pathology) data [14]. Comparison of the expression level of *MMP1* and *MMP3* between human hepatic stellate cell line LX-2 and Huh-7 cells, they are highly expressed in LX-2 cells compared with Huh-7 cells (data not shown). Although the amounts of *MMP1* and *MMP3* are low in hepatocytes compared with hepatic stellate cells, hepatocytes acquire MMPs expression after arsenite exposure, while control culture conditions do not express MMPs. This suggests that arsenite exposure alters the microenvironment of the tumor, making it more malignant and susceptible to invasion. Although p53 is traditionally known as a tumor supprressor by means of inducing apoptosis and senescence [26], recent study has shown that activation of hepatocyte p53 creates a microenvironment prone to tumor formation from hepatic progenitor cells [16]. Combined with this finding and cellular senescence has an aspect related to the promotion of tumorigenesis via SASP, it is suggested that p53 expression transiently suppresses tumorigenesis by inhibiting cell proliferation, but in the long term, remaining senescent cells play a role in promoting tumorigenesis through SASP.

Among the SASP factors we investigated in the current study, in addition to MMP1 and MMP3, overexpression of MMP10 has been reported to promote invasion, metastasis in head and neck cancer, and tongue cancer [27–29]. In HCC, MMP10 has also been reported as contributing to HCC development, participating in tumor angiogenesis, growth, and dissemination [30]. GDF15, growth differentiation factor 15, is reported to induce immunosuppression via CD48 on regulatory T cells in HCC [31], and is reported to induce dysfunction of NK cells [32]. Since inhibition of NK cells has been reported to induce promotion of HCC [33] and regulatory T cells are associated with a poor prognosis in HCC [34], induction of GDF15 in hepatocytes may contribute to the function of senescent cells as promoters of tumorigenesis. PAI-1 is a fibrinolysis inhibitor involved in regulating protein degradation and homeostasis while assisting wound healing [35]. In HCC, PAI-1 favors the angiogenic switch [36]. In terms of angiogenesis, VEGFA is the major factor for angiogenesis that binds to two tyrosine kinase (TK) receptors, VEGFR-1 (Flt-1) and VEGFR-2 (KDR/Flk-1), and regulates endothelial cell proliferation, migration, vascular permeability, secretion and other endothelial functions [37, 38]. In HCC, VEGF is frequently expressed [39] and the expression is elevated in multiple tumor types and correlates with the prognosis of tumor patients [40]. IL-6 production is known as an essential factor in inducing HCC in the mice model and the production level is different between sexes [41]. Autocrine IL-6 contributes to HCC progenitor cell to HCC progression [42]. Therefore, increase of those SASP factors can contribute to tumorigenesis by arsenic exposure.

We have shown that arsenite exposure induces premature senescence followed by SASP factors in both hepatic stellate cells [14] and hepatocytes. Which premature senescence of hepatocytes or hepatic stellate cells has a more significant effect or whether they have synergistic effects on carcinogenesis remains unclear. Additionally, hepatocyte senescent cells have the potential to affect hepatic stellate cells. To our knowledge, however, there are no reports that investigate the senescent hepatocytes which affect hepatic stellate cells. Further studies are needed on those points.

Previously, both tumor-suppressive and tumor-progressive effects have been reported as playing the role of hepatocyte senescence in hepatocellular carcinoma [15–17, 33, 43]. Since immune response plays an important role in determining whether cellular senescence followed by SASP factors act as tumor suppressors or promoters [17, 33, 43, 44], further studies are needed about the interaction between arsenite exposure-induced SASP factors and immune response. In addition, we focused on stress-induced premature senescence in the present study and did not investigate telomeres. However, a recent report showed that telomeric 8-oxo-guanine (8oxoG), typically induced by oxidative stress, is sufficient to trigger p53-dependent senescence. Although acute 8oxoG production does not shorten telomeres, it instead generates fragile sites and mitotic DNA synthesis at telomeres, indicating impaired replication [45]. Therefore, further studies are needed to determine whether arsenite exposure induces this type of telomere dysfunction.

In recent years, there has been active development of senolytic drugs that selectively eliminate senescent cells for the treatment of age-related diseases, including cancer [46–48]. In fact, studies have reported the suppression of cancer progression when senescent cells in hepatocytes were eliminated [15, 17]. This suggests that senolytic drugs could potentially be an effective approach for combating liver cancer induced by arsenite exposure. To identify molecular targets for senolytic drugs for arsenite induced senescent cells that could play a role in future treatment strategies, additional research is required to understand the mechanisms underlying arsenite-induced senescence and the persistence of senescent cells even after discontinuation of arsenite exposure.

## 5. Conclusions

**Fig. 8:**
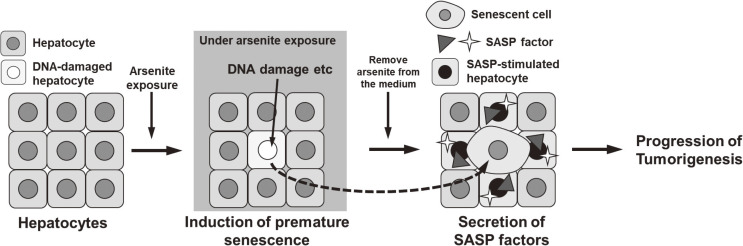
Proposed mechanism involving premature senescence of hepatocytes in malignancy of arsenite exposure-induced hepatic cancer

Arsenite exposure induces premature senescence in hepatocyte-derived cells and increases SASP factors that are related to hepatic tumorigenesis. Once arsenite exposure induces premature senescence, the senescent cells remain even after cessation of exposure (Fig. 8).

## Abbreviations

SA-β-gal: senescence-associated β-galactosidase
SASP: senescence-associated secretory phenotype
HCC: hepatocellular carcinoma
FBS: fetal bovine serum

## Supplementary information

**Additional file 1:** **Table S.1** Primer sequences for real-time PCR.

## Declarations

### Availability of data and material

All data are incorporated into the article and its online supplementary material.
