# Supplementary material for: Arsenite exposure induces premature senescence and senescence-associated secretory phenotype (SASP) in human hepatocyte-derived cell line Huh-7
Source: Environ Health Prev Med. 2024 Dec 27;29:74. doi: 10.1265/ehpm.24-00139 (PMC11701098; doi:10.1265/ehpm.24-00139)
Supplement: Supplementary file 1 — Additional file 1: Table S.1 Primer sequences for real-time PCR. [file ehpm-29-074-s001.docx]

Table S.1

Primer sequences for real-time PCR

A)　Measurement of gene expression levels

| Gene | 5’ Primer (5’-3’) | 3’ Primer (5’-3’) | Annealing temperature (°C) |
| --- | --- | --- | --- |
| *P21* | GGGCTGGGAGTAGTTGTCTT | AGCCGAGAGAAAACAGTCCA | 68 |
| *LAMINB1* | TGACAGTATCCCGAGCATCC | TCGCCTCTGATTCTTCCACA | 64 |
| *MMP1* | AGGCAAGTTGAAAAGCGGAG | CATCTGGGCTGCTTCATCAC | 64 |
| *MMP3* | TGGCCATCTCTTCCTTCAGG | TAGGGTGTGGATGCCTCTTG | 64 |
| *MMP10* | CCAAGAGGCATCCATACCCT | CTCCAGTATTTGTCCGCTGC | 64 |
| *GDF15* | CCGAAGACTCCAGATTCCGA | GGTGTTCGAATCTTCCCAGC | 64 |
| *PAI-1* | TGTACAAGGAGCTCATGGGG | CGGAACAGCCTGAAGAAGTG | 64 |
| *VEGFA* | TTGGGGAGCTTCAGGACATT | CTGAATCTTCCAGGCAGTGC | 64 |
| *IL-6* | GCTGCAGGACATGACAACTC | CCCATGCTACATTTGCCGAA | 64 |
| *18S rRNA* | TACCACATCCAAGGAAGGCAG | TGCCCTCCAATGGATCCTC | 64 |
| *RPLP1* | ATTTTCCCTGCCACCATTGC | CCCCAGTGCAGTTTTCAACA | 64 |
